# Anatomical Guidelines and Technical Tips for Neck Aesthetics with Botulinum Toxin

**DOI:** 10.1055/s-0044-1788284

**Published:** 2024-08-09

**Authors:** Hyewon Hu, Soo-Bin Kim, Jovian Wan, Lisa Kwin Wah Chan, Alvin Kar Wai Lee, Olena Sydorchuk, Arash Jalali, Mariana César Corrêa, Jong-Seo Kim, Kyu-Ho Yi

**Affiliations:** 1 Division in Anatomy and Developmental Biology, Department of Oral Biology, Human Identification Research Institute, BK21 FOUR Project, Yonsei University College of Dentistry, Seoul, Korea; 2 Asia Pacific Aesthetic Academy, Hong Kong; 3 EverKeen Medical Centre, Hong Kong; 4 Wlabel, Seoul, Korea; 5 One Clinic MD, Vancouver, Canada; 6 Clinic Dr Mariana Correa, Brazil; 7 Kimjongseo Plastic Surgery Clinic, Seoul, Korea; 8 Maylin Clinic (Apgujeong), Seoul, Korea

**Keywords:** neck aesthetics, botulinum toxin, beautiful neck, trapezius muscle, submandibular gland, platysma muscle

## Abstract

Botulinum toxin can be used for various purposes to enhance neck aesthetics, addressing concerns such as platysmal bands, optimizing the cervicomental angle, preventing worsening of horizontal neckline and decolletage lines during aging, submandibular gland hypertrophy, and hypertrophied superior trapezius muscle. Understanding the anatomy of muscles such as the trapezius, platysma, and submandibular gland is crucial for achieving desirable outcomes with botulinum toxin administration. Techniques for injecting botulinum toxin into these muscles are discussed, emphasizing safety and efficacy. Specific injection points and methods are detailed for treating platysmal bands, optimizing the cervicomental angle, addressing submandibular gland hypertrophy, and managing hypertrophied superior trapezius muscle. Careful consideration of anatomical landmarks and potential complications is essential for successful botulinum toxin injections in these areas.

## Introduction

**Fig. 1:**
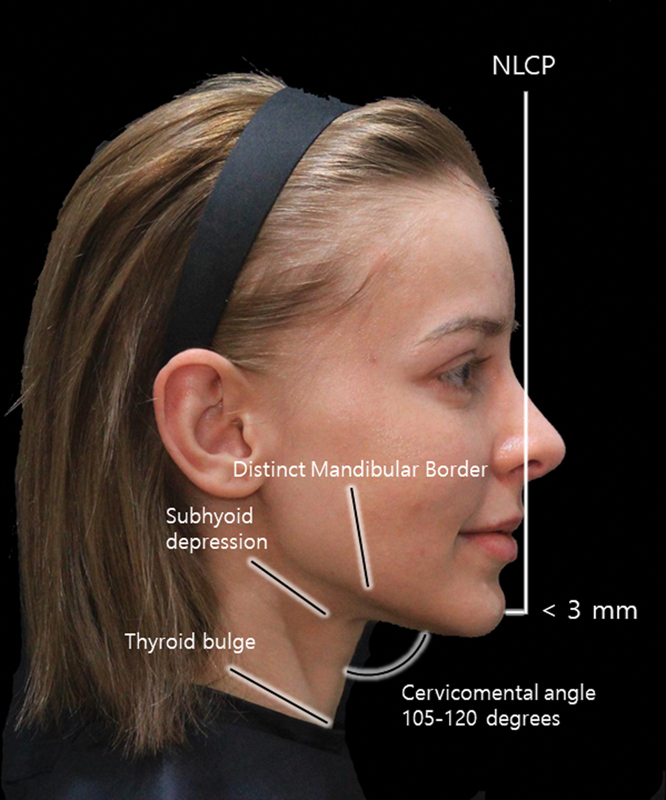
The figure exhibits an illustrative representation elucidating the cervicomental angle, highlighting characteristics emblematic of an ideal neck contour. The illustration showcases a distinct delineation of the mandibular border, shaping an angle falling within the spectrum of 105 to 120 degrees.

**Fig. 2:**
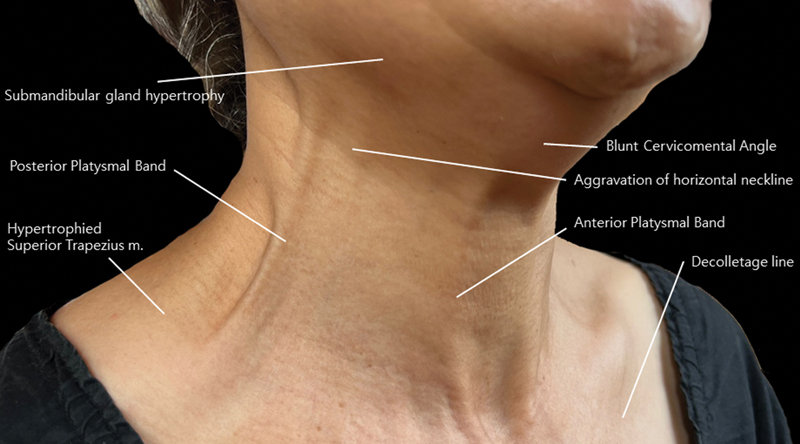
This figure illustrates the potential applications of botulinum toxin administration, which may encompass addressing various conditions including submandibular gland hypertrophy, platysmal bands, hypertrophied superior trapezius muscle, optimization of the cervicomental angle, and prevention of the exacerbation of horizontal neckline and decolletage line during the aging process.

Age-related changes in the neck region can manifest as the accumulation of submental fat, decreased skin firmness leading to a reduced cervicomental angle, bone resorption, and the development of jowls, ultimately resulting in compromised neck contours. 1 2 The concept of a “youthful neck” typically denotes a well-defined cervicomental angle ranging from approximately 105 to 120 degrees, ideally forming a 90-degree angle along the submental line ( Fig. 1 ). Botulinum toxin offers a versatile approach to attaining a more youthful neck appearance. Its application can target various concerns including platysmal bands, optimizing the cervicomental angle, preventing the exacerbation of horizontal neck and décolletage lines with age, submandibular gland hypertrophy, and hypertrophied superior trapezius muscle 3 4 5 ( Fig. 2 ). Attaining an aesthetically pleasing neck appearance necessitates a comprehensive understanding of the pertinent musculature, encompassing the trapezius, platysma muscle, and submandibular gland, approached from an anatomical standpoint. This article explores the anatomical features of these structures and investigates the effects of botulinum toxin administration on them. Informed consent was obtained from all cases involved in the article.

## Platysma Muscle

**Fig. 3:**
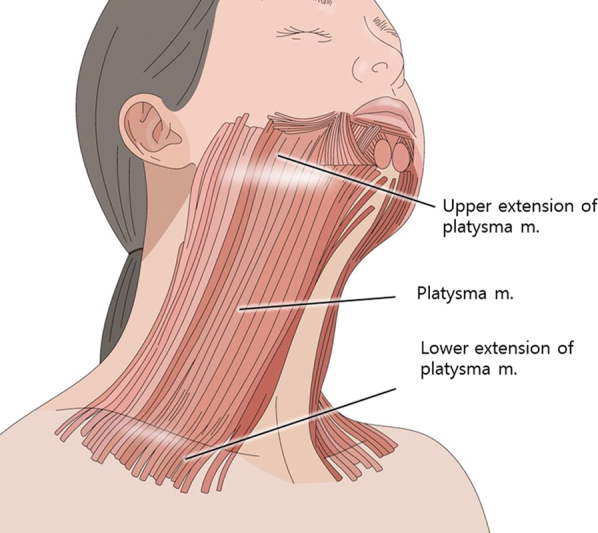
The figure depicts the origin of the platysma muscle from the fascial structure of the pectoralis major and anterior deltoid muscles. It extends downward toward the lower face integrating smoothly with the superficial musculoaponeurotic system.

**Fig. 4:**
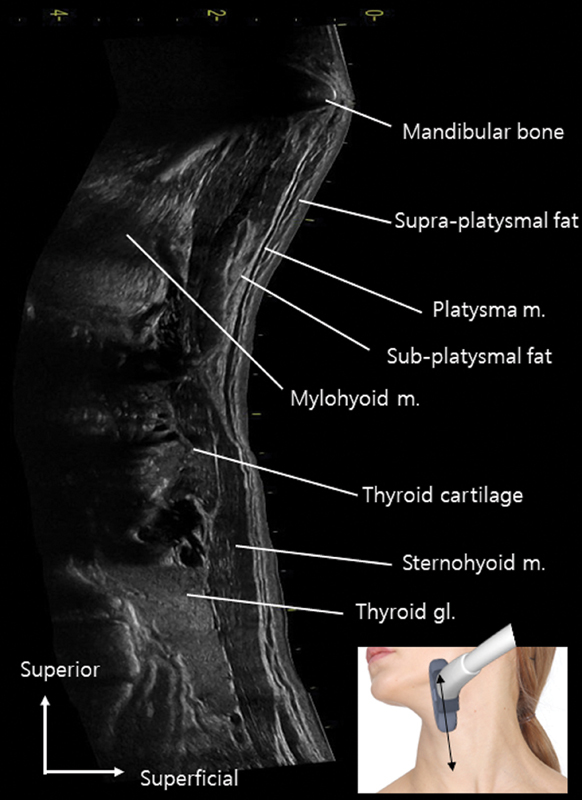
The image presents a panoramic view of ultrasonography, showcasing a longitudinal image captured from the midpoint of the mandibular border.

**Fig. 5:**
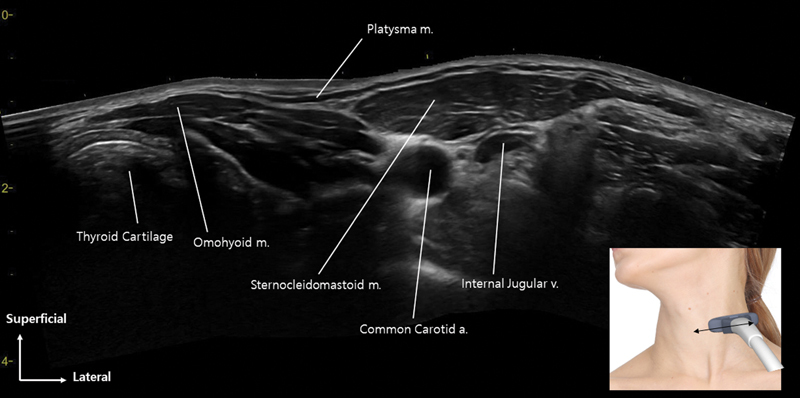
The figure displays a panoramic view of ultrasonography, illustrating a horizontal image obtained from the midpoint between the mandibular border and clavicle.

The platysma muscle, located superficially in the neck region, occupies a significant anatomical space and plays a crucial role in facial expression dynamics. Its superior segment facilitates the downward movement of the cheeks and the widening of the lips, while the inferior segment contributes to the elevation of neck skin 6 7 8 ( Figs. 3 – 5 ). Originating from the fascial connections of the pectoralis major and anterior deltoid muscles, the platysma muscle extends inferiorly towards the lower face, integrating with the superficial musculoaponeurotic system (SMAS). Variability exists in its distribution, with some individuals lacking its presence in the lower middle neck area, exhibiting partial presence in the upper region, and displaying fibers crossing the submental area. In aesthetic terminology, it is commonly referred to as the superficial cervical fascia, encompassing adipose tissue, lymph nodes, nerves, and vessels. 9

The platysma muscle partially overlies the sternocleidomastoid muscle, with the interstitial space filled by the superficial cervical fascia, comprising fibroareolar connective tissue, while the deep cervical fascia lies beneath. 8 10 11 12 13 14

Yi et al utilized ultrasonography to investigate the anatomical characteristics of the platysma muscle and its role in the formation of platysmal bands. Their investigation, involving 24 participants exhibiting neck bands, conducted ultrasonographic assessments in both resting and contracted states, supplemented by examinations on cadaveric specimens to measure muscle thickness at various locations. Their findings revealed that during contraction, the anterior and posterior borders of the platysma muscle exhibited increased thickness compared to the middle portion, forming a convex contour. However, muscle thickness remained relatively constant at rest. Contraction predominantly influenced the medial and lateral margins, particularly in posterior bands, suggesting that interventions targeting platysmal bands should concentrate on the muscle's anterior and posterior borders. 5 11

**Fig. 6:**
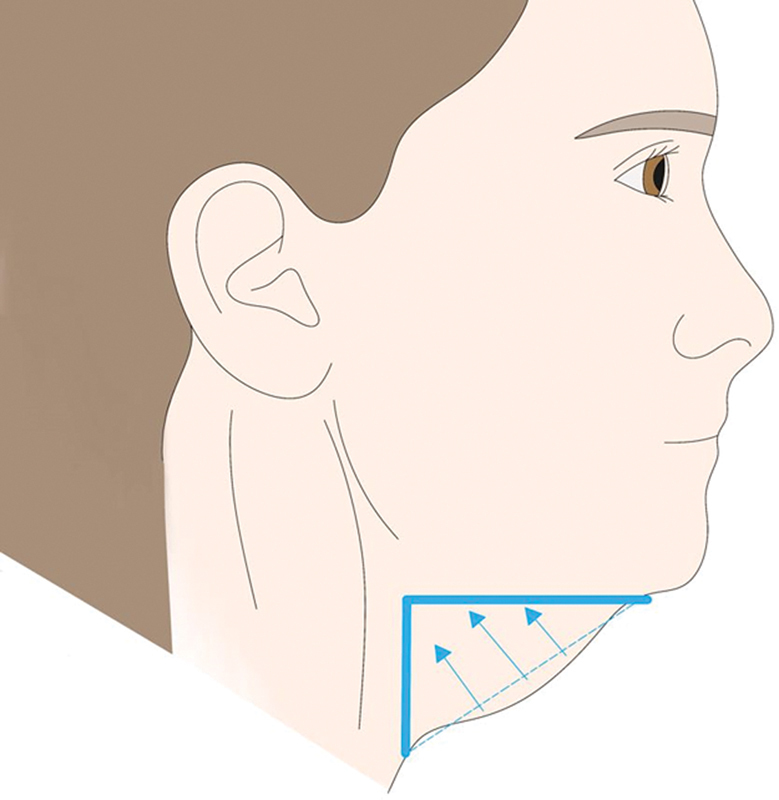
The illustration of the phenomenon where the platysma muscle contracts, resulting in a wider cervicomental angle and contributing to neck sagging. Upon injection of botulinum toxin into the platysma muscle, relaxation occurs, leading to the creation of a more curved neckline when viewed laterally.

Botulinum toxin represents the primary nonsurgical modality for addressing platysmal bands and serves as a postoperative adjunct to minimize the likelihood of band recurrence. Typically, botulinum toxin injections yield favorable outcomes, particularly in the management of vertical platysmal bands. 15 However, horizontal lines on the neck, which are congenital, exhibit less responsiveness to botulinum toxin treatment in comparison to vertical bands. Nevertheless, the management of horizontal necklines is feasible through temporary paralysis of the platysma muscle, which often exerts downward traction on the lower face and subcutaneous neck tissue. 16 Additionally, aging-related contracture of the platysma muscle may result in a cervicomental angle exceeding 120 degrees. Botulinum toxin injections can optimize this angle and prevent exacerbation of the horizontal neckline ( Fig. 6 ). Moreover, platysma muscle contraction in the lower region contributes to the appearance of décolletage lines around the sternal region, which can also be ameliorated through botulinum toxin injections. Numerous authors have proposed diverse injection techniques for the platysma muscle to mitigate signs of aging, advocating specific injection sites tailored to individual indications. 4 17 18 19 20 21

## Platysmal Band

### Assessment

Before administering the injection to assess the platysmal band, direct the patient to turn their head to one side, raise their chin, and reveal their lower teeth. This evaluation should be performed twice, once for each side, holding the muscle bands with your nondominant hand while injecting the botulinum toxin directly into them.

### Injection Points and Tips

**Fig. 7:**
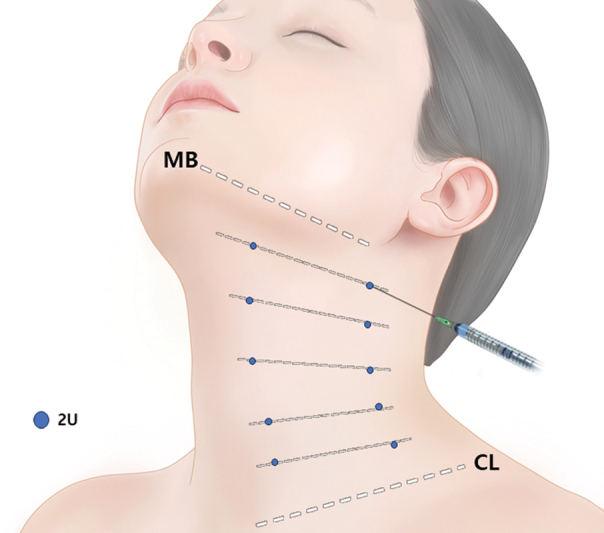
The figure demonstrates the injection technique for botulinum toxin, where 2 units (U) are administered per point. Five points are injected along each band, with a spacing of approximately 2 cm between each injection site. A total of 40 units are utilized for both the anterior and posterior bands. To prevent paralysis of swallowing muscles and potential dysphagia, as well as to avoid affecting the sternocleidomastoid muscle, intradermal or subdermal injection is recommended. CL, clavicular line; MB, mandibular border.

Each injection point receives 2 units of Letibotulinum toxin type A, with five injection points per band spaced approximately 1 inch apart. Evaluation of platysmal bands is crucial, commencing with patient instruction to create a melancholy facial expression by vigorously pulling down on the corners of the mouth to hyperactivate the platysma muscle. Subsequently, botulinum toxin is injected directly into the muscular bands, targeting five points ranging from below the jawline to the clavicle ( Fig. 7 ). To minimize the risk of inadvertent effects on neighboring anatomical structures, intradermal or subdermal injection is advised to prevent the paralysis of swallowing muscles, potentially leading to dysphagia, and to avoid affecting the sternocleidomastoid muscle.

### Complications

Bruising is the most commonly encountered adverse reaction. If blood vessels are ruptured, immediate pressure application for 5 minutes is advised. Excessive dosage can sometimes lead to dysphonia, dysphagia, and weakness in neck muscles as a result of botulinum toxin spreading into underlying muscles. Xerostomia may occur due to botulinum toxin diffusion into the submandibular gland. Dysphonia typically involves a “high-tone disturbance.” Diffusion of botulinum toxin into neck supporting muscles can result in difficulty controlling neck movement. Additionally, patients with cervical neck disk issues may experience worsened symptoms due to weakened neck support muscles.

## Horizontal Neckline Alleviation and Cervicomental Angle

### Assessment

Most patients who attend the clinic with concerns regarding horizontal necklines exhibit noticeable symptoms before any formal assessment. To conduct a comprehensive evaluation of horizontal necklines, it is essential to instruct the patient to intentionally retract their chin, with the goal of producing a distinct double chin.

### Injection Points and Tips



**Video 1** Demonstration of treating platysmal bands with injection points.


**Fig. 8:**
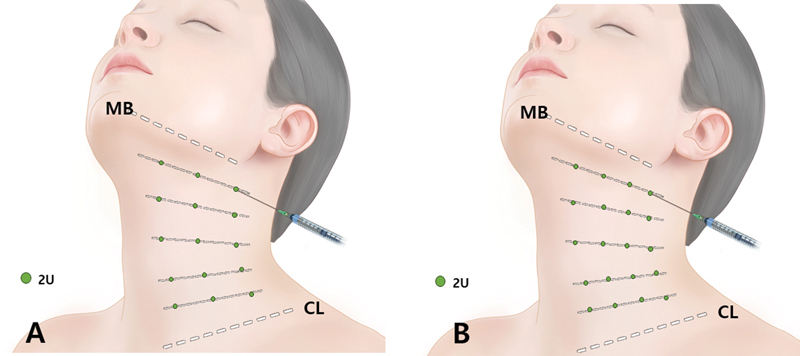
The figure illustrates the option for additional injections in the middle portion of the platysma muscle, apart from the platysmal band injection points. One ( **A** ) to two ( **B** ) more vertical lines can be targeted for injection, with each point receiving 2 units (U) of botulinum toxin. The total units administered may range from 60 to 80 units depending on individual patient factors. An intradermal or subdermal injection is recommended for these additional injections. CL, clavicular line; MB, mandibular border.

**Fig. 9:**
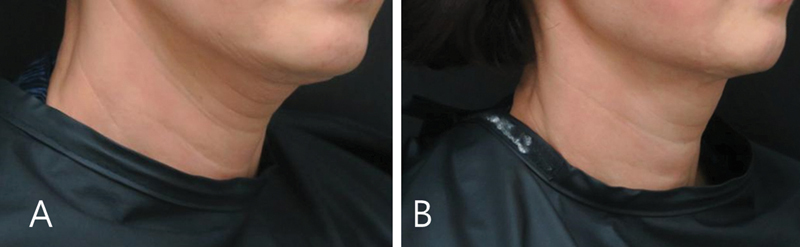
The figure depicts the case of a 45-year-old woman who underwent two cycles of botulinum toxin injections for jawline lifting and alleviation of the horizontal neckline. The injections were administered at a 3-month interval between each cycle. The photographs show the before ( **A** ) and after ( **B** ) treatment outcomes from a 45-degree viewing angle.

In addition to targeting the injection points for the platysmal bands, the central area of the platysma muscle can also be injected with one to two additional vertical lines. Each injection point is administered 2 units of botulinum toxin ( Fig. 8 ). The total dosage may vary between 60 and 80 units, depending on the specific needs of the individual patient. For safety and effectiveness, intradermal or subdermal injection is recommended. This approach is particularly advantageous for patients seeking improvement in horizontal necklines and enhancement of the cervicomental angle ( Fig. 9 ). A practical demonstration of the injection technique is available in Video 1 .

### Complications

Complications associated with botulinum toxin injections into the platysma muscle include ecchymosis/hematoma, which commonly arises owing to the superficial positioning of the extrajugular vein and anterior cervical vein, rendering them prone to injury during injection. A previous study documented a 5% incidence of mild dysphagia and a 1% incidence of neck weakness subsequent to botulinum toxin injections. 17

## Jawline (Nefertiti) Lift

### Injection Points and Tips



**Video 2** Demonstration of treating jawline lifting.


**Fig. 10:**
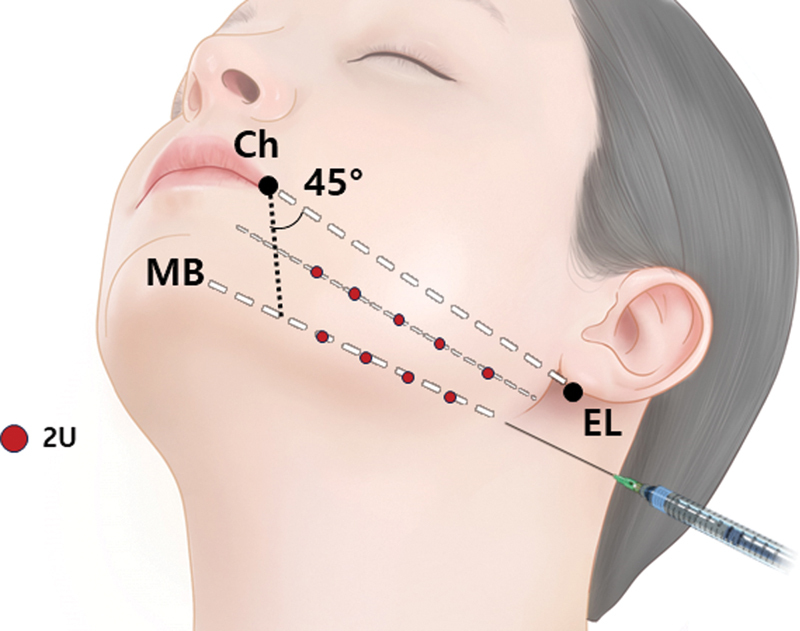
The figure demonstrates the technique for jawline lifting using botulinum neurotoxin injections. Two units (U) are administered per point, totaling 20 U of botulinum neurotoxin per side. The injections are performed in two lines, spaced at a distance of two finger widths. One line is positioned directly above the mandibular lower border (MB), while the other line is placed below the line connecting the cheilion (Ch) and ear lobule (EL).

**Fig. 11:**
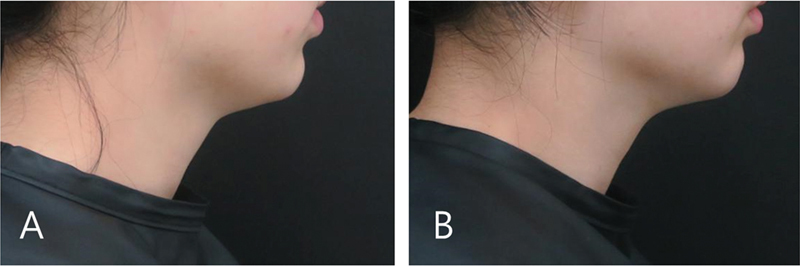
The figure presents the case of a 36-year-old woman undergoing botulinum toxin treatment for horizontal neckline alleviation and cervicomental angle optimization. The treatment involved a single session of botulinum toxin injection. The photographs depict the before ( **A** ) and 3 months after ( **B** ) outcomes of the botulinum toxin treatment from a lateral view.

To enhance the jawline, each injection site receives 2 units of botulinum toxin. A total of 20 units of botulinum neurotoxin (BoNT) are administered on each side. The injections are conducted along two lines: one positioned directly above the lower border of the mandible, and another below the line connecting the corner of the mouth (cheilion) and the earlobe. Each line consists of five injection sites spaced 2 cm apart. To prevent diffusion into the depressor labii inferioris muscle, injections along the lower border of the mandible should be delivered above the marionette line, at approximately a 45-degree angle from the corner of the mouth. Injection of BoNT for jawline elevation is recommended at the subdermal or intradermal levels ( Fig. 10 ). The patient's case involves jawline elevation and cervicomental angle injection ( Fig. 11 ). A practical demonstration of the injection technique is provided in Video 2 .

### Complications

When administering injections for jawline treatment, it is vital to ensure accurate placement to prevent the activation of muscles such as the risorius, depressor anguli oris, or depressor labii inferioris muscles, which could cause an asymmetric smile. Prior assessment of patients is necessary to determine if the risorius muscle is activated during smiling, and injections should be avoided in such cases.

## Submandibular Gland

**Fig. 12:**
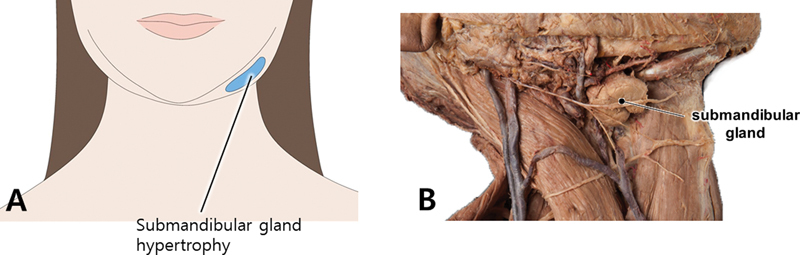
The figure illustrates the bulky appearance of the jawline resulting from hypertrophy of the submandibular gland ( **A** ). Additionally, it includes a dissected image ( **B** ) showing the lower border of the mandible in cadaveric dissection, where the platysma muscle and cervical fascia have been removed.

Botulinum toxin is commonly utilized to target the submandibular gland, with the aim of reducing the visibility of its irregular jawline contour. This treatment serves both aesthetic and functional purposes, particularly in the management of hypersalivary diseases ( Fig. 12 ). 22 Situated within the submandibular triangle, the submandibular gland ranks as the second largest among the three major salivary glands. While it typically contributes approximately 70% of saliva at rest, its secretion diminishes in favor of the parotid gland when stimulated. In the treatment of excessive salivation using botulinum toxin, injections are directed toward both the parotid and submandibular glands. 23 24 25

Given its proximity to swallowing muscles and its smaller volume relative to the parotid glands, accessing the submandibular gland for botulinum toxin injections poses challenges. Consequently, only a fraction of BoNT is typically administered into the submandibular gland. The administration of botulinum toxin injections for submandibular gland treatment entails the risk of complications, including dysphagia and bruising. These complications often arise due to the passage of facial vessels over the gland and the presence of the lingual nerve beneath it, underscoring the importance of considering anatomical factors during the procedure. 26

Adverse outcomes may result if botulinum toxin is inadvertently injected into muscles other than the targeted submandibular gland. To mitigate such risks, precise injections should be directly administered into the submandibular gland based on a comprehensive understanding of anatomy, with treatment initiated using a conservative BoNT dosage. Additionally, careful consideration should be given to the potential impact of repeated short-term BoNT injections, which may trigger antibody production and compromise treatment efficacy.

Recent anatomical studies have identified specific injection sites within certain muscles guided by external anatomical landmarks. In a study conducted by Lee et al, the primary aim was to delineate the anatomical landmarks of the submandibular gland to facilitate BoNT injections. 27 Investigating 34 submandibular glands from 20 cadavers, Lee et al established a designated safety zone for injecting botulinum toxin into the submandibular gland, positioned between 20 and 35% from the mandible angle when viewed from below. Additionally, it should be located 1.5 cm below the inferior line of the mandible, with the needle inserted to a depth of 2.0 cm from the skin surface. While their findings generally corresponded with our study, 14 specimens deviated from their specified range in terms of accuracy. 28

### Assessment

The submandibular gland can be located effectively by using a vertical line intersecting the lateral canthus as a guide. Following this line from the lateral canthus, gently place the index finger beneath the mandibular border and palpate the mass, pulling posteriorly while assessing the tactile sensation.

### Injection Points and Tips



**Video 3** Demonstration of treating submandibular gland injection with botulinum neurotoxin.


**Fig. 13:**
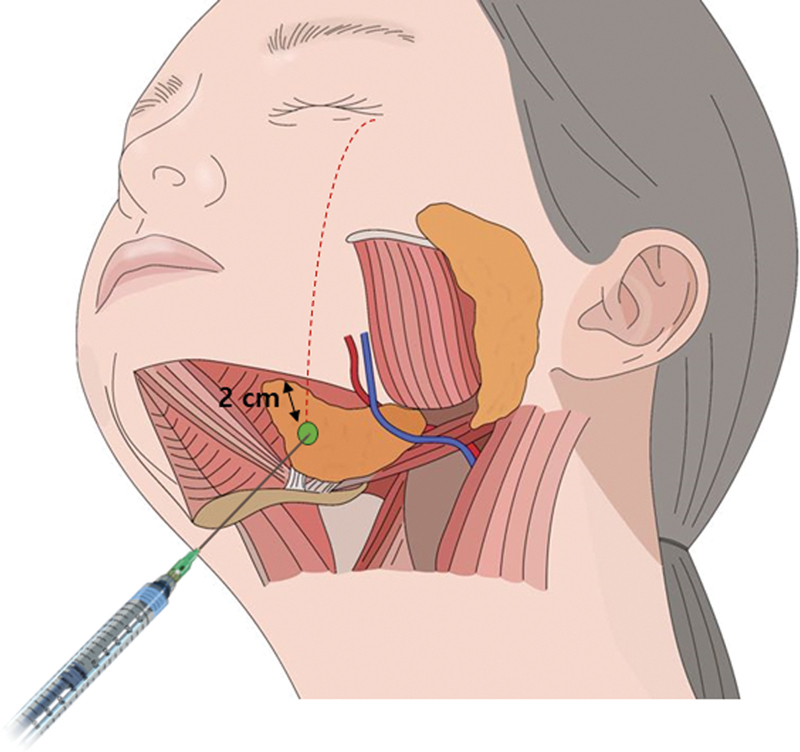
The figure depicts the ideal injection points for the submandibular gland. These points are located where a vertical line intersects the lateral canthus and aligns approximately 2 cm away from the inferior border of the mandible. This location is considered optimal for injection as there are no facial arteries or veins running over it, minimizing the risk of complications.

It is advisable to utilize a 0.5-inch needle for intrasubmandibular injections. Targeting the vertical line intersecting the lateral canthus as the injection point ensures safety, even when positioned at the anterior portion of the submandibular gland, which represents approximately one-third of the medial side of the gland. This location helps avoid potential vascular damage. Additionally, it is essential to exercise caution when injecting immediately below the mandibular border, as it may pose risks to vascular structures. Therefore, it is recommended to inject at a distance of 2 cm from the border to minimize such risks (see Fig. 13 ), 29 particularly in the absence of ultrasound guidance. The dosage of botulinum toxin units for injections depends on the size of the submandibular gland, typically ranging from 15 to 30 units per side. Letibotulinum toxin type A is administered per injection point, with one injection point. The botulinum toxin is diluted with 2.5 mL of normal saline to achieve a concentration of 4 U/0.1 mL (refer to Video 3 ).

During intraglandular injections, it is crucial to observe any pain sensations reported by the patient upon needle insertion and botulinum toxin injection. It is important to verify that the patient experiences this sensation during the injection process.

### Complications

Administering botulinum toxin injections for submandibular gland treatment may result in complications such as dysphagia and bruising, given the passage of facial vessels over the gland and the presence of the lingual nerve beneath it. These complications often occur due to a lack of awareness of the anatomical factors involved in the procedure. 26 Undesirable outcomes can occur if the botulinum toxin is inadvertently injected into muscles other than the targeted submandibular gland. To prevent such effects, precise injections should be directly administered into the submandibular gland based on anatomical understanding, and treatment should commence with a low dose of botulinum toxin.

## Trapezius Muscle

Body contouring with botulinum toxin often targets specific muscle groups, including the superior trapezius, triceps brachii, quadriceps, and gastrocnemius muscles. 3 30 31 32 33 34 35 36 37 Among these, the superior trapezius muscle is frequently the focus of botulinum toxin injections. 38 An enlarged superior trapezius muscle can create the impression of a shorter neck and overly muscular shoulders, which may be a cosmetic concern, particularly among women in the general population. This enlargement can pose challenges when wearing off-the-shoulder clothing that exposes the shoulders and neckline. Originating from the occipital protuberance, nuchal ligament, and ligamentum nuchae of C7–T12 vertebrae, the trapezius muscle inserts on the lateral third of the clavicle, acromion process, and spine of the scapula. Comprising superior, middle, and inferior portions, its primary functions include posture maintenance and assistance in scapular movements. Motor innervation is predominantly provided by the spinal accessory nerve. 39

Zhou et al 40 evaluated the aesthetic outcomes of a single botulinum toxin injection for bilateral hypertrophy of the superior trapezius muscles. While most participants experienced satisfactory body contouring results, 7% did not show significant improvement, possibly due to factors such as incorrect injection sites or insufficient dosage.

Bae et al 41 documented a considerable reduction in muscle thickness of the superior trapezius following a single botulinum toxin injection. The hyperactivity of the superior trapezius muscle is often associated with lifestyle factors such as prolonged use of computers and smartphones in hunched postures, 42 43 44 45 leading not only to aesthetic concerns but also to the development of shoulder pain over time.

**Fig. 14:**
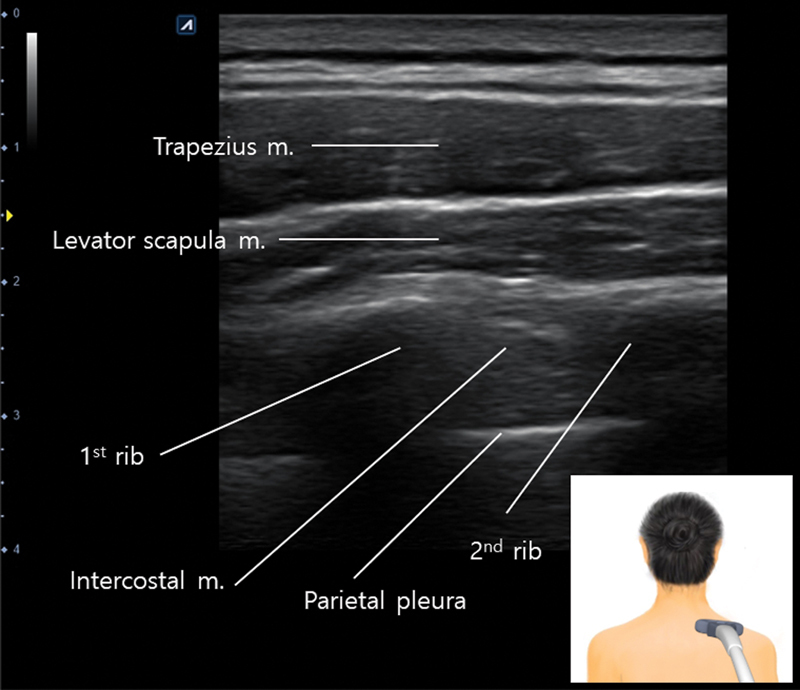
The figure presents an ultrasonographic observation of the superior trapezius muscle. The depth from the epidermis to the rib measures approximately 2 cm in this instance. Therefore, a needle length of 0.5 inch would be appropriate for targeting the trapezius muscle.

Previous studies have employed botulinum toxin injections into the trapezius muscle, with dosages ranging from 10 to 50 units and targeting one to five injection sites. 40 41 46 47 48 Salavati et al 49 reported that the thickness of the superior trapezius muscle ranged from 10 to 13 mm ( Fig. 14 ), gradually decreasing to 3 to 6 mm in the lower trapezius region. Botulinum toxin typically diffuses up to 2 to 4 cm from the injection site. 50 To mitigate overspreading, multiple injections may be considered.

Yi et al 3 employed a modified Sihler method to investigate intramuscular neural distribution within the trapezius muscles, analyzing 16 specimens. They identified distinct neural distribution patterns in the superior trapezius muscles, with the highest arborization observed at specific horizontal and vertical fractions within each muscle region. Botulinum toxin injections should be administered at these arborized sections. For the upper trapezius, the most arborized pattern was located horizontally from 1/5 to 2/5 and vertically from 2/10 to 4/10. Care should be taken to avoid injecting the area horizontally from 2/5 to 3/5 and vertically from 2/10 to 4/10, corresponding to the nerve entry point, to prevent nerve damage. Potential complications, such as pneumothorax resulting from the shallow depth from the skin surface to the rib cage, and bilateral contraction of the superior trapezius leading to nerve compression due to compensatory hypertrophy of deeper extensor muscles, should be considered by health care providers during injection administration.

## Hypertrophied Trapezius

### Assessment

A well-developed trapezius muscle can create the illusion of a shorter neck and less aesthetically pleasing shoulder contours, while also contributing to the perception of a relatively larger face. Extended periods of slouching over electronic devices, such as computers or smartphones, can exacerbate the contraction of the trapezius muscle, leading to muscle hypertrophy, as well as potential occurrences of myalgia and migraines. It is crucial to inquire about these factors from the patient before proceeding with treatment. This is why botulinum toxin injections into the trapezius muscle are considered for migraine management. To assess the thickness and sensitivity of the superior trapezius, instruct the patient to shrug their shoulders while gently pinching the muscle between their thumb and index finger.

### Injection Points and Tips



**Video 4** Demonstration of treating upper trapezius injection with botulinum neurotoxin.


To address wrinkles, a standard dosage of 100 units of botulinum toxin is mixed with 2.5 mL of normal saline, resulting in a concentration of 4 U/0.1 mL. Conversely, for body contouring purposes, the same 100 units are dissolved in a larger volume of 5 mL, comprising 4.8 mL of normal saline and 0.2 mL of epi-lidocaine, resulting in a concentration of 2 U/0.1 mL. This increased reconstitution volume aids in the effective diffusion of botulinum toxin into the expansive muscles, while the addition of 0.2 mL of epi-lidocaine (1:100,000 epinephrine) impedes the rapid dispersion of botulinum toxin into the systemic circulation via the muscle's rich vascular network. Typically, a 0.5-inch (1.25 cm) 30-gauge needle is used for these procedures.

**Fig. 15:**
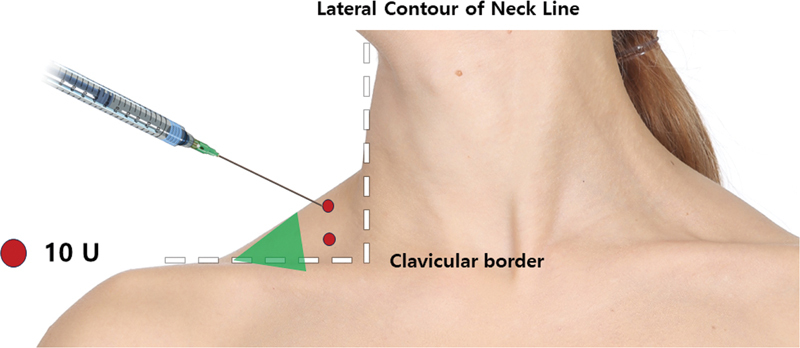
The figure outlines the recommended approach for administering botulinum toxin injections to the upper trapezius muscle. Injection should be targeted at each arborized section, with particular emphasis on the most arborized pattern located lateral to the contour of the neckline. Imaginary lines drawn from the frontal view, aligning with the lateral contour of the neckline and clavicular border, aid in identifying the injection sites. The authors suggest injecting in the medial half to avoid potential damage to the accessory spinal nerve, which enters at this point (highlighted in green). A total of 10 units of letibotulinum toxin are used, with each injection point receiving 10 units.

**Fig. 16:**
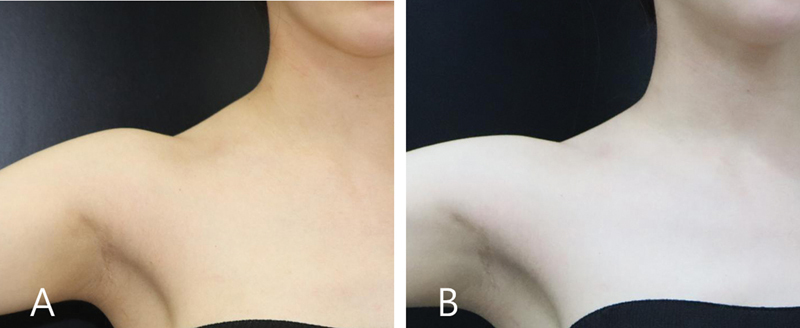
The figure illustrates the case of a 34-year-old woman presenting with a chief complaint of hypertrophied trapezius. She underwent one session of botulinum toxin therapy. Panel ( **A** ) displays the condition before the treatment, while panel ( **B** ) shows the outcome 3 months after the treatment.

Botulinum toxin administration should target each arborized section within the upper trapezius muscle. The most arborized pattern is situated lateral to the contour of the neckline. For precise targeting, imaginary lines are drawn from the lateral contour of the neckline to the clavicular border when viewed from the frontal perspective. These areas should be the focus for botulinum toxin injection. The authors recommend targeting the medial half for injection, as the lateral half carries a risk of damaging the accessory spinal nerve (highlighted in green), which enters at this point. The authors utilized 10 U of Letibotulinum toxin, with each injection point receiving 10 units (refer to Fig. 15 ). The patient case involves a hypertrophied superior trapezius muscle, depicted before and after treatment ( Fig. 16 ). A practical demonstration of the injection technique is provided in Video 4 .

### Complications

Discomfort due to muscle weakness is a common complication, often resolving within 2 months. It is crucial to adjust the dosage based on the muscle size to minimize this effect. Additionally, mild adverse symptoms such as stiffness and a feeling of heaviness may occur. However, more severe complications like pneumothorax have been reported in patients receiving injections, especially with deep injections, often linked to the inherent lack of precision in blind techniques. 51 Furthermore, injury to the spinal accessory nerve trunk can result in unilateral trapezius palsy. 52

## Conclusion

Age-related changes in the neck area, such as the accumulation of submental fat and decreased skin firmness, often result in undesirable aesthetic effects like reduced cervicomental angles and the emergence of jowls. Botulinum toxin injections present a versatile solution for addressing various concerns associated with aging neck appearance, including platysmal bands, optimizing cervicomental angles, and minimizing horizontal neckline and decolletage lines ( Table 1 ). A comprehensive understanding of the anatomical characteristics of relevant structures, such as the platysma muscle, is crucial for achieving optimal outcomes with botulinum toxin treatments.

Injection techniques targeting platysmal bands involve precise placement of botulinum toxin to effectively mitigate signs of aging. Strategic injections along the middle portion of the platysma muscle can also aid in alleviating the horizontal neckline and optimizing cervicomental angles. Additionally, injections for jawline lifting can enhance overall neck appearance by targeting specific points along the mandibular border.

Complications associated with botulinum toxin injections into the platysma muscle, such as ecchymosis/hematoma and dysphagia, highlight the importance of careful injection techniques and patient assessment to minimize adverse effects. Similarly, administering botulinum toxin injections to the submandibular gland necessitates attention to anatomical considerations to avoid complications like dysphagia and bruising.

Injections targeting the superior trapezius muscle aim to address aesthetic concerns related to muscle hypertrophy, especially in individuals with shortened necks and prominent shoulders. A thorough understanding of the anatomical distribution of nerves within the trapezius muscle is crucial for avoiding adverse effects such as nerve damage and pneumothorax.

Overall, botulinum toxin injections offer a valuable nonsurgical option for achieving a youthful neck appearance, provided they are administered with precision and consideration of anatomical factors. Collaborative efforts between healthcare providers and patients are essential for tailoring treatment approaches to individual needs and achieving optimal aesthetic outcomes.

**Table 1:** The table provides a concise summary of key injection points

Facial region	Injection technique and points	Typical complications
Platysmal band	It starts with the patient's head turned to one side and chin up. Inject 2 units of botulinum toxin at five points per band, from below the jawline to the clavicle, spaced about an inch apart. Target the anterior and posterior borders	Bruising is common Excessive dosage can cause dysphonia, dysphagia, and neck muscle weakness
Horizontal neckline and cervicomental angle	Additional injections in the central platysma muscle; 2 units per injection point vertically along the neck. Total dosage of 60 to 80 units depending on patient needs.	Bruising and potential dysphagia from affecting swallowing muscles, should avoid sternocleidomastoid muscle to prevent complications
Jawline (Nefertiti) lift	2 units of botulinum toxin per injection along two lines: one above and one below the mandible, spaced 2 cm apart, avoiding depressor muscles for symmetry	Misplacement can activate risorius or depressor muscles causing an asymmetric smile
Submandibular gland	Use a 0.5-inch needle for injections, targeting the gland via a vertical line intersecting the lateral canthus; 15 to 30 units per side, injected intraglandularly to avoid surrounding muscular structures.	Bruising and dysphagia if botulinum toxin spreads to swallowing muscles. Proper anatomical knowledge is crucial to avoid complications
Trapezius muscle	Dosages range from 10 to 50 units targeting one to five sites across the muscle to address hypertrophy and migraine symptoms. The most arborized sections of the muscle are targeted to avoid nerve damage	Discomfort, stiffness, potential pneumothorax, and risk of unilateral trapezius palsy if the spinal accessory nerve is damaged
